# Draft genomes of six *Streptomyces* species from a United States biogeography survey

**DOI:** 10.1128/mra.00996-25

**Published:** 2025-12-08

**Authors:** Janani Hariharan, Nicole M. Feriancek, James R. Doroghazi, Mallory J. Choudoir, Peter Diebold, Ashley N. Campbell, Kevin Panke-Buisse, Daniel H. Buckley

**Affiliations:** 1 School of Integrative Plant Science, Cornell University 5922, Ithaca, New York, USA; University of Wisconsin-Madison, Madison, Wisconsin, USA

**Keywords:** *Streptomyces*, novel species, biogeography, secondary metabolite

## Abstract

*Streptomyces* bacteria play key ecological and functional roles in terrestrial ecosystems. We surveyed soil samples across the continental United States, identifying six novel *Streptomyces* species. Here, we report the whole genome sequences of these strains and their predicted biosynthetic products, providing additional information for studying biological and chemical diversity in this ubiquitous species.

## ANNOUNCEMENT

Representatives of the genus *Streptomyces*, phylum Actinomycetota*,* are found in a wide range of habitats and are common in soils worldwide. Filamentous actinomycetes are responsible for producing over two-thirds of known antibiotics, with *Streptomyces* producing roughly 80% of these compounds (1). Genetic and genomic diversity within the genus, as well as patterns of antibiotic production, can diverge along geographical and elevational gradients (2–5). *Streptomyces* strains play important ecological and functional roles in ecosystems, including the breakdown of complex carbon substrates like cellulose and chitin (6, 7). The genus *Streptomyces* thus has important applications for agriculture and medicine.

We conducted a survey of soil samples across the continental United States to further understand the biogeography and genetic diversity of this group, which resulted in the identification of several novel species.

We describe six such species in this study, which are drawn from a culture collection of over 1,000 strains. The sampling location of each strain can be seen in Table 1.

**TABLE 1:** Sampling locations and genome assembly statistics of the *Streptomyces* species isolated in this study^*a*^

Strain name	Closest named relative (% ANI)	Accession no.	Sampling location	Latitude	Longitude	No. of scaffolds	Total length (Mbp)	Scaffold N50 (bp)	Median coverage (×)	GC content (%)	% completeness	% contamination
CO7	S. fragilis NBRC 12862^T^ (93.9)	GCA_053474865.1	Mancos, Colorado	37.32° N	−108.14° W	234	6.75	626,773	30	73.0	99.46	0.64
ME109	Streptomyces sp. WAC00263 (88.3)	GCA_008042115.1	Kennebunk, Maine	43.4° N	−70.54° W	302	9.13	493,345	53	71.7	100	2.21
MS191	S. exfoliatus DSM 41,693^T^(86.1)	GCA_008042125.1	Starkville, Mississippi	33.46° N	−88.8° W	261	7.32	388,359	41	72.5	99.91	1.7
OR43	S. laculatispora DSM 42090^T^ (88.9)	GCA_008042275.1	Astoria, Oregon	46.18° N	−123.85° W	140	8.75	4,652,464	83	70.9	99.62	1.17
T39	S. zhihengii DSM 42176^T^(92.1)	GCA_008042045.1	Austin, Texas	30.2° N	−97.77° W	292	7.67	1,120,392	203	72.9	99.37	2.08
UW30	Streptomyces sp. KS_5 (91.9)	GCA_008042075.1	Troy, North Carolina	35.71° N	−79.88° W	193	9.45	1,367,314	51	70.7	99.68	0.79

^*a*^ Average nucleotide identity (ANI) was calculated using fastANI (8) against validly named reference species according to the International Council of Nomenclature for Prokaryotes (ICNP).

The strains were isolated using a standard plate dilution method and grown on glycerol-arginine agar supplemented with cycloheximide as described previously (9). After 7–14 days of aerobic incubation at 25°C, colonies were transferred and purified on glycerol-arginine agar and maintained as spore stocks in 20% glycerol suspensions (vol/vol) at −80°C. DNA was extracted from liquid cultures grown in yeast extract-malt extract medium at 30°C for 7 days using a modified phenol-chloroform extraction protocol optimized for *Streptomyces* as outlined in (10). We verified that each isolate belonged to the *Streptomyces* genus by obtaining the full-length 16S rRNA gene sequence via Sanger sequencing using primers 27F and 1,492R as described in (11). Extracted DNA was quantified using the PicoGreen assay (Thermo Fisher Scientific, MA, USA) prior to library preparation and barcoding with the Nextera XT kit (Illumina, Inc., CA, USA) according to the manufacturer’s instructions. These libraries were then sequenced on a MiSeq machine using the v3 kit (2×300 bp reads) at the Biotechnology Resource Center, Cornell University. A total of 20,757,936 reads were generated across all samples.

Raw reads were assembled using the A5-miseq pipeline with default trimming and assembly parameters (12). Contigs generated at this step were assembled into scaffolds using MeDuSa (13). CO7 was assembled with SPAdes v3.12 (14) and scaffolded with RagTag (15). Draft assemblies were evaluated for completeness and contamination with CheckM (16). Assembly statistics, including sequencing coverage, are provided in Table 1. Genome annotation of scaffolds was done using Prokka v1.14.6 (17). Default parameters were used for all software.

**Fig 1:**
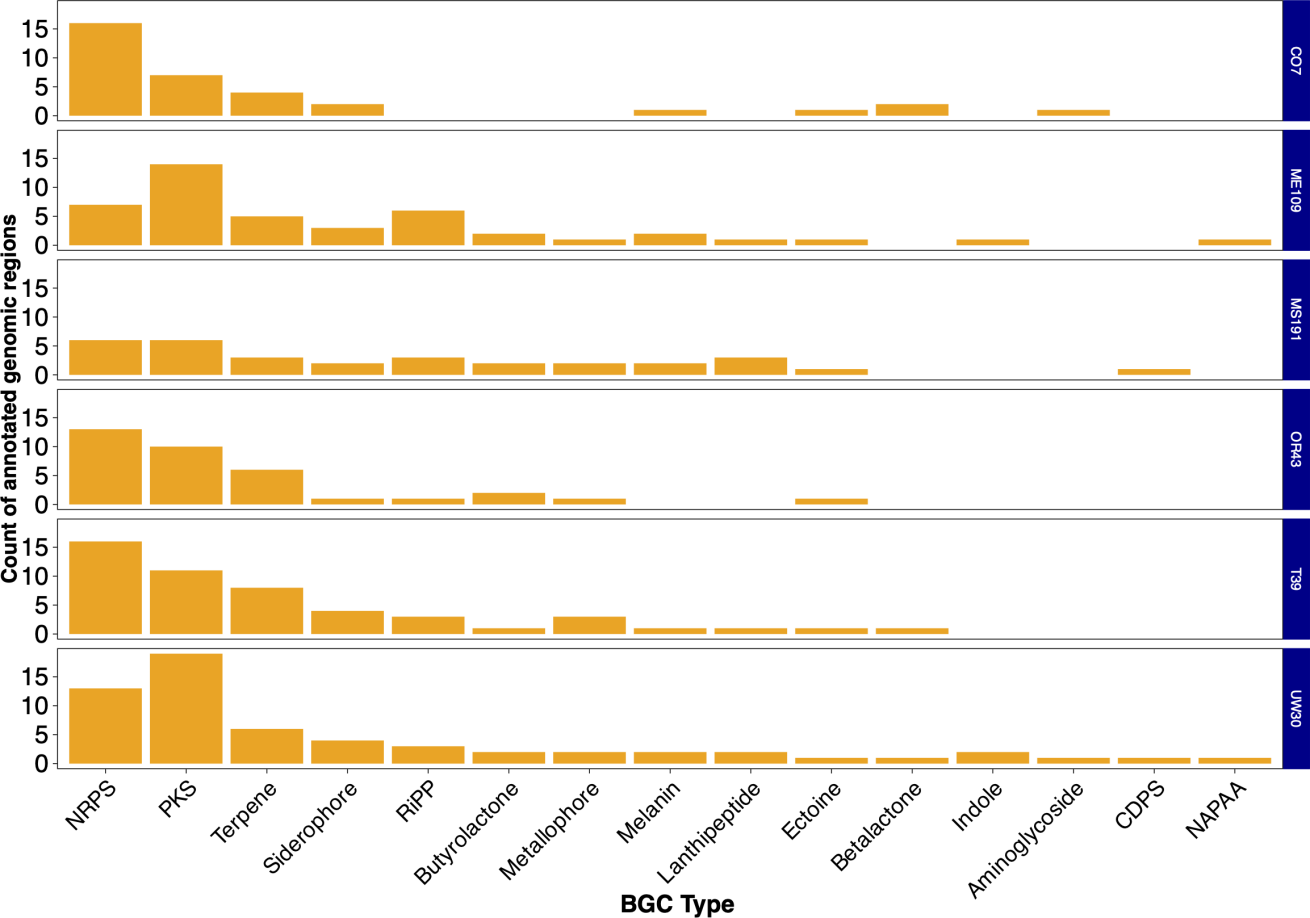
Distribution of BGCs among the six genomes sequenced in this study. NRPS, non-ribosomal peptide synthase; PKS, polyketide synthase domains; RiPP refers to precursors of ribosomally synthesized and post-translationally modified peptides. CDPS stands for tRNA-dependent cyclodipeptide synthase, and NAPAA refers to non-alpha poly-amino acid.

The average genome size was 8.2 Mbp, with a minimum of 6.75 Mbp and a maximum of 9.45 Mbp. The average N50 value was 1.44 Mbp (range 0.39–4.65 Mbp). Each genome contained a rich array of biosynthetic gene clusters (BGCs) as identified using antiSMASH v7.0 (18). The average number of BGCs per genome was 44±13 . We found terpene and siderophore clusters in each genome, while other BGC types were only found in some genomes (Fig. 1). BGCs that were only detected in one strain are not shown: bacteriocins (CO7), phenazine, aminopolycarboxylic acid, thioamide-containing domains, lasso peptides, β-lactam clusters (UW30), and an aryl polyene cluster (MS191).

## Data Availability

The genome assemblies and raw reads for all strains are available under BioProject accession number PRJNA496211. Accession numbers for genome assemblies are GCA_008042115.1 (ME109), GCA_008042125.1 (MS191), GCA_008042275.1 (OR43), GCA_008042045.1 (T39), GCA_008042075.1 (UW30), and GCA_053474865.1 (CO7). The strains have been deposited in the JCM or NRRL culture collections under the following accession numbers: NRRL B-65526 (ME109), JCM 33243/NRRLB-65525 (MS191), JCM 33237/NRRL B-65527 (OR43), JCM 33238/NRRL B-65532 (T39), and NRRL B-65530 (UW30).
